# Contribution of modifiable risk factors for hypertension and type-2 diabetes in Peruvian resource-limited settings

**DOI:** 10.1136/jech-2015-205988

**Published:** 2015-08-06

**Authors:** Antonio Bernabé-Ortiz, Rodrigo M Carrillo-Larco, Robert H Gilman, William Checkley, Liam Smeeth, J Jaime Miranda, CRONICAS Cohort Study Group

**Affiliations:** 1 CRONICAS Center of Excellence in Chronic Diseases, Universidad Peruana Cayetano Heredia, Lima, Peru; 2 Department of International Health, Bloomberg School of Public Health, Johns Hopkins University, Baltimore, USA; 3 Área de Investigación y Desarrollo, AB PRISMA, Lima, Peru; 4 Division of Pulmonary and Critical Care, School of Medicine, Johns Hopkins University, Baltimore, USA; 5 Faculty of Epidemiology and Population Health, London School of Hygiene and Tropical Medicine, London, UK; 6 Department of Medicine, School of Medicine, Universidad Peruana Cayetano Heredia, Lima, Peru

**Keywords:** DIABETES, Epidemiology of chronic non communicable diseases, HYPERTENSION

## Abstract

**Background:**

It is important to understand the local burden of non-communicable diseases including within-country heterogeneity. The aim of this study was to characterise hypertension and type-2 diabetes profiles across different Peruvian geographical settings emphasising the assessment of modifiable risk factors.

**Methods:**

Analysis of the CRONICAS Cohort Study baseline assessment was conducted. Cardiometabolic outcomes were blood pressure categories (hypertension, prehypertension, normal) and glucose metabolism disorder status (diabetes, prediabetes, normal). Exposures were study setting and six modifiable factors (smoking, alcohol drinking, leisure time and transport-related physical activity levels, TV watching, fruit/vegetables intake and obesity). Poisson regression models were used to report prevalence ratios (PR). Population attributable risks (PAR) were also estimated.

**Results:**

Data from 3238 participants, 48.3% male, mean age 45.3 years, were analysed. Age-standardised (WHO population) prevalence of prehypertension and hypertension was 24% and 16%, whereas for prediabetes and type-2 diabetes it was 18% and 6%, respectively. Outcomes varied according to study setting (p<0.001). In multivariable model, hypertension was higher among daily smokers (PR 1.76), heavy alcohol drinkers (PR 1.61) and the obese (PR 2.06); whereas only obesity (PR 2.26) increased the prevalence of diabetes. PAR showed that obesity was an important determinant for hypertension (15.7%) and type-2 diabetes (23.9%).

**Conclusions:**

There is an evident heterogeneity in the prevalence of and risk factors for hypertension and diabetes within Peru. Prehypertension and prediabetes are highly prevalent across settings. Our results emphasise the need of understanding the epidemiology of cardiometabolic conditions to appropriately implement interventions to tackle the burden of non-communicable diseases.

## Introduction

Worldwide, non-communicable diseases (NCDs) cause 36 million deaths annually,^1^ and are responsible for 54% of disability-adjusted life years.^2^ In one study, more than 60% of deaths from NCDs were attributable to four preventable cardiometabolic factors, with high-blood pressure having the largest effect.^1^ Moreover, high-blood pressure continues to be the leading risk factor for mortality, whereas mortality burden due to glucose metabolism disorders, specifically type-2 diabetes, almost doubled between the years 1980 to 2010.^1^

A modelling study reported that reducing the prevalence of six risk factors, including smoking and alcohol consumption, among others, would contribute to reducing NCD mortality due to cardiovascular disease and diabetes, with most of these benefits in low and middle-income countries (LMICs).^3^ From a resource-constrained setting perspective, these findings point out that attention to well-established modifiable risk factors need to be addressed to reduce the negative burden of hypertension and type-2 diabetes on mortality, as well as their impact on organisation and costs of healthcare delivery systems.^4^ ^5^

Health research and development efforts for NCDs in LMICs are limited, despite repeated calls for action.^6^ Thus, for purposes of establishing priority, it is important to understand the local burden of disease including within-country and between-country heterogeneity of NCD profiles and their risk factors. This would provide evidence to set suitable interventions along with appropriate goals and targets.^2^ On this information, strategies could be defined at the local and regional level. For example, on the basis of global-scale policy scenarios, considering mortality benefits and feasibility, reductions in tobacco are recommended as a ‘best buy’ initiative.^3^ Yet, within the Latin American region, Peru shows much lower smoking rates,^7^ indicating that although smoking prevention is needed in general, global ‘best buy’ policies require local scrutiny and adaptation to maximise larger preventative gains.

The aims of this study were to characterise the profile of two leading cardiometabolic conditions, hypertension and type-2 diabetes, across distinct Peruvian geographical settings. In addition, emphasis was placed on the magnitude of the associations between these cardiometabolic conditions and modifiable risk factors in these settings, their respective population attributable risks (PAR) and the pattern of aggregation of common risk factors.

## Methods

### Study design and setting

A cross-sectional, baseline assessment of the CRONICAS cohort study^8^ was conducted between the years 2010 and 2011 in four Peruvian settings that differed by level of urbanisation, altitude and degree of household and environmental pollution: Pampas de San Juan de Miraflores in Lima, a highly-urban setting; Puno, a high altitude area located at 3830 m above sea level, contributing with urban and rural sites; and Tumbes, a semiurban coastal location where rural farming and fishing villages have become intermixed with rapidly-growing urban areas. Detailed description of the four study sites is provided elsewhere.^8^

### Participants

Individuals aged ≥35 years, full-time residents in the area, who provided informed consent, were invited to participate in the study. We identified a sex- and age-stratified random sample (35–44, 45–54, 55–64 and ≥65 years) of potentially eligible subjects. Single stage random sampling was conducted using the most updated census available (year 2010) in each of the sites. Only one participant per household was enrolled.

### Study variables

Main outcomes were two: blood pressure status and glucose metabolism disorder status. Blood pressure status was categorised based on hypertension and prehypertension definitions. Hypertension was defined as any of the following conditions: systolic blood pressure (SBP) ≥140 mm Hg or diastolic blood pressure (DBP) ≥90 mm Hg; or self-report of physician diagnosis and current use of antihypertensive drugs^9^; whereas prehypertension was defined as a systolic pressure from 120 to 139 mm Hg or a diastolic pressure from 80 to 89 mm Hg. On the other hand, glucose metabolism disorder status was generated based on diabetes and prediabetes definitions. Diabetes was defined as any of the following conditions: fasting glucose ≥126 mg/dL; self-report of physician diagnosis and currently receiving antihyperglycaemic medications; whereas prediabetes was defined as fasting plasma glucose values from 100 to 125 mg/dL.^10^

The main exposures were study site (Lima, urban Puno, rural Puno and Tumbes, see settings above), and six well-established modifiable factors associated with hypertension and diabetes: daily smoking (≥1 cigarette/day, self-report), heavy alcohol drinking (2 or more nights of alcohol intake in the past month and having ever drunk 6 or more drinks at a time), leisure time and transport-related physical activity based on the International Physical Activity Questionnaire domains as recommended for Latin American populations^11^), number of hours watching TV (defined as 2 or more hours watching TV per day during weekdays, self-report), fruit and vegetable intake (minimum consumption recommended by World Health Organisation, ie, 5 or more servings of fruits or vegetables per day^12^) and obesity (using traditional body mass index (BMI) traditional cutoffs^13^).

Other variables of interest included in the analysis were self-reported parental history of hypertension and/or diabetes, and metabolic syndrome defined by using the 2009 harmonised definition that incorporates region-specific cut-off points.^14^ Sociodemographic variables were also included as potential confounders including sex, age (35–44, 45–54, 55–64 and 65+ years), education level (<7 years, 7–11 years and ≥12 years), and socioeconomic status using a wealth index based on assets and household facilities divided into tertiles.

### Procedures

Participants responded to a detailed questionnaire. Fieldworkers in rural areas were fluent in Spanish, Aymara or Quechua and they administered the survey to those with poor literacy. Fieldworkers measured weight, height and blood pressure in triplicate using standardised techniques.^9^ Fasting blood samples were obtained and analysed in a single facility, and the quality of assays was checked with regular external standards and internal duplicate assays were monitored by BioRad (http://www.biorad.com). Plasma glucose was measured using an enzymatic colourimetric method (GOD-PAP, Modular P-E/Roche-Cobas, Germany). Detailed information on evaluation and measurement techniques is reported elsewhere.^8^

### Statistical analysis

Statistical analyses were conducted in STATA V.13 (Stata Corp, College Station, Texas, USA) in 2014. Characteristics of the population were tabulated according to study site and comparisons were performed using one-way analysis of variance or Kruskal-Wallis test for numerical variables, and χ^2^ test for categorical variables. age-standardised prevalence of our outcomes of interest was calculated using WHO standard population distribution.^15^

Poisson regression with robust SEs^16^ was conducted to compare blood pressure status (prehypertension vs normal blood pressure, and hypertension vs normal blood pressure) and glucose metabolism status (prediabetes vs normal glucose levels, and diabetes vs normal glucose levels) according to study setting. Four different models were built. Model 1 was the crude model, only including the outcome and the exposure. Model 2 also included age, sex, education level and socioeconomic status. Model 3 included the previous variables and also parental history of hypertension/diabetes accordingly. Finally, model 4, in addition, included obesity status. Poisson regression was also used to determine the strength of association between our six modifiable risk factors and our outcomes of interest, controlling for age, sex, education level, socioeconomic status and study site. Participants aware of their hypertension or diabetes diagnosis were excluded from these models accordingly. For all the regression models, prevalence ratios (PR) and 95% confidence intervals (95% CI) were calculated.

In addition, population attributable risk (PAR) was calculated using the *punaf* command in STATA^17^, where estimations are performed using the recommendation of Greenland and Drescher for cross-sectional studies.^18^ Finally, calculations of the cumulative effect of risk factors—adding common risk modifiable factors—on our outcomes of interest were performed controlling for potential confounders and results are presented graphically using a Forest plot.

### Ethics

All participants provided verbal informed consent due to high-illiteracy rates, especially in rural areas. The study was approved by the Institutional Review Boards at Universidad Peruana Cayetano Heredia and AB PRISMA, in Lima, Peru, and at the Bloomberg School of Public Health, Johns Hopkins University, in Baltimore, USA.

## Results

### Participant characteristics

Overall response rate after enrolment was 62.9% (4325/6872) and, of these, 83.3% (3601/4325) completed all questionnaires. Among those with completed questionnaires, 89.8% (3232/3601) and 87.1% (3135/3601) had all clinical and blood laboratory evaluations completed, respectively (see online supplementary E-figure S1).

A total of 3238 participants, 48.3% male, mean age 45.3 years (IQR: 45.3–65.2), were included in the analysis. Of them, 21.3% had ≥12 years of education and 90.3% had a family income of <US$550 per month, indicating the predominance of low socioeconomic status among participants. Detailed population characteristics as total and by study setting are provided in table 1.

**Table 1:** Participant characteristics: comparisons according to study site

	Total	Lima	Urban Puno	Rural Puno	Tumbes	
	N=3238	n=1052	n=574	n=581	n=1031	p-value*
Sociodemographics—n (%)						
Male	1565 (48.3%)	506 (48.1%)	277 (48.3%)	270 (46.5%)	512 (49.7%)	0.67
Age, years—median (IQR)	55.1 (45.3; 65.2)	54.8 (45.5; 64.7)	55.7 (45.3; 65.3)	55.9 (45.8; 65.9)	55.0 (44.9; 65.0)	0.34
Education level						
<7 years	1492 (46.1%)	454 (43.2%)	91 (15.8%)	374 (64.1%)	573 (55.6%)	<0.001
7–11 years	1056 (32.6%)	416 (39.6%)	156 (27.2%)	172 (29.5%)	312 (30.3%)	
≥12 years	690 (21.3%)	181 (17.2%)	327 (57.0%)	37 (6.4%)	145 (14.1%)	
Socioeconomic Status (tertiles)						
Lowest	1021 (31.5%)	127 (12.1%)	135 (23.5%)	419 (72.1%)	340 (33.0%)	<0.001
Middle	1107 (34.2%)	387 (36.8%)	155 (27.0%)	147 (25.3%)	418 (40.5%)	
Highest	1110 (34.3%)	538 (51.1%)	284 (49.5%)	15 (2.6%)	273 (26.5%)	
Lifestyles—n (%)						
Daily smoking	103 (3.2%)	34 (3.2%)	12 (2.1%)	1 (0.2%)	56 (5.4%)	<0.001
Heavy alcohol drinking	172 (5.3%)	58 (5.5%)	37 (6.5%)	17 (2.9%)	60 (5.8%)	0.03
Leisure time and transport-related physical activity						
Lowest	1031 (31.9%)	201 (19.1%)	122 (21.3%)	149 (25.6%)	559 (54.2%)	<0.001
Moderate	1784 (55.1%)	637 (60.6%)	372 (65.0%)	360 (61.9%)	415 (40.3%)	
Highest	421 (13.0%)	213 (20.3%)	78 (13.6%)	73 (12.5%)	57 (5.5%)	
Hours watching TV (2+ hours per day)	1382 (42.7%)	509 (48.4%)	265 (46.2%)	83 (14.3%)	525 (50.9%)	<0.001
Fruits and vegetables intake (5+ servings per day)	132 (4.1%)	70 (6.7%)	38 (6.6%)	11 (1.9%)	13 (1.3%)	<0.001
Parental history—n (%)						
Hypertension among at least one of the parents	683 (21.1%)	192 (18.3%)	120 (20.9%)	25 (4.3%)	346 (33.6%)	<0.001
Diabetes among at least one of the parents	238 (7.4%)	74 (7.0%)	32 (5.6%)	3 (0.5%)	129 (12.5%)	<0.001
Anthropometric measurements—n (%)						
Body mass index						
Normal weight (BMI <25 kg/m^2^)	953 (29.5%)	243 (23.2%)	135 (24.0%)	317 (54.5%)	255 (24.7%)	<0.001
Overweight (BMI ≥25–29.9 kg/m^2^)	1409 (43.6%)	471 (45.0%)	278 (49.4%)	206 (35.4%)	450 (43.7%)	
Obesity (BMI ≥30 kg/m^2^)	869 (26.9%)	332 (31.7%)	150 (26.6%)	59 (10.1%)	326 (31.6%)	
Clinical Profile						
SBP (mm Hg)—mean (SD)	117.6 (19.1)	117.4 (18.8)	112.2 (17.5)	116.5 (17.3)	121.5 (20.3)	<0.001
DBP (mm Hg)—mean (SD)	73.4 (11.1)	72.8 (11.2)	71.8 (10.1)	75.2 (10.1)	74.0 (11.8)	<0.001
Fasting glucose (mg/dL)—mean (SD)	98.2 (33.9)	97.7 (33.2)	96.6 (32.4)	90.2 (21.3)	103.6 (39.4)	<0.001
A1c (%)—mean (SD)	6.0 (1.2)	5.9 (1.1)	6.0 (1.1)	5.9 (0.8)	6.2 (1.4)	<0.001
Cardiometabolic condition—n (%)						
Blood pressure						
Normal	1799 (55.4%)	589 (56.0%)	394 (68.6%)	327 (56.3%)	481 (46.7%)	<0.001
Prehypertension	809 (24.9%)	251 (23.9%)	101 (17.6%)	182 (31.3%)	273 (26.5%)	
Hypertension	641 (19.7%)	212 (20.1%)	79 (13.8%)	72 (12.4%)	277 (26.9%)	
Glucose metabolism disorder						
Normal	2313 (74.2%)	767 (74.4%)	395 (76.7%)	473 (87.5%)	678 (65.7%)	<0.001
Prediabetes	588 (18.8%)	207 (20.1%)	83 (16.1%)	51 (9.4%)	247 (24.0%)	
Diabetes	217 (7.0%)	57 (5.5%)	37 (7.2%)	17 (3.1%)	106 (10.3%)	
Metabolic syndrome	1465 (47.0%)	505 (49.0%)	246 (47.9%)	150 (27.7%)	564 (54.8%)	<0.001

*p-values were calculated comparing study sites using one-way analysis of variance or Kruskal–Wallis test for numerical variables, and χ^2^ test for categorical variables.

A1c, glycated haemoglobin; BMI, body mass index; DBP, diastolic blood pressure; SBP, systolic blood pressure. Results may not add up due to missing values.

### Prevalence of hypertension's and diabetes's awareness, treatment and control and their association with study setting

Age-standardised prevalence of prehypertension and hypertension was 23.7% (95% CI 22.3% to 25.1%) and 16.2% (95% CI 15.1% to 17.3%), respectively. Prevalence rates of these conditions varied according to study setting (p<0.001), but there was no uniform pattern: prehypertension was frequent in urban Puno, but hypertension was more frequent in semiurban Tumbes (table 1). Of the 641 cases of hypertension, 392 (61.3%) individuals were aware of their diagnosis, 318/392 (81.1%) reported to be on treatment and only 129/318 (40.6%) had controlled blood pressure levels.

Similarly, age-standardised prevalence of prediabetes and diabetes was 18.1% (95% CI 16.9% to 19.5%) and 6.2% (95% CI 5.5% to 7%), respectively. Prediabetes and diabetes rates were both higher in the semiurban setting (Tumbes) compared to the others (p<0.001). Of the 217 cases of type-2 diabetes, 133 (61.3%) individuals were aware of their diagnosis, 95/133 (71.4%) reported to be on treatment and 60/95 (63.2%) had controlled blood glucose levels.

### Association between cardiometabolic outcomes and study site

Results of the multivariable models did not show a clear pattern of association between study setting and the outcomes of interest: Tumbes, the semiurban setting, had higher prevalence of prehypertension, hypertension, prediabetes and diabetes, when compared to Lima. In addition, urban Puno had lower prehypertension and hypertension rates when compared to Lima, whereas rural Puno had greater prevalence of prehypertension but not of hypertension and lower prevalence of prediabetes but not diabetes (table 2).

**Table 2:** Hypertension and diabetes according to study setting: bivariable and multivariable models using multinomial logistic regression

	Model 1	Model 2	Model 3	Model 4
	PR (95% CI)	PR (95% CI)	PR (95% CI)	PR (95% CI)
Prehypertension (vs normal)				
Lima	1 (Reference)	1 (Reference)	1 (Reference)	1 (Reference)
Urban Puno	0.68 (0.56 to 0.84)	0.64 (0.52 to 0.79)	0.64 (0.52 to 0.79)	0.64 (0.52 to 0.79)
Rural Puno	**1.20 (1.03 to 1.40)**	**1.20 (1.01 to 1.43)**	**1.22 (1.02 to 1.45)**	**1.28 (1.07 to 1.53)**
Tumbes	**1.21 (1.05 to 1.39)**	**1.24 (1.07 to 1.42)**	**1.19 (1.03 to 1.38)**	**1.18 (1.02 to 1.36)**
Hypertension (vs normal)				
Lima	1 (Reference)	1 (Reference)	1 (Reference)	1 (Reference)
Urban Puno	**0.63 (0.50 to 0.80)**	**0.59 (0.47 to 0.75)**	**0.61 (0.48 to 0.77)**	**0.57 (0.44 to 0.73)**
Rural Puno	**0.68 (0.54 to 0.86)**	**0.68 (0.53 to 0.88)**	**0.73 (0.56 to 0.94)**	0.81 (0.62 **to** 1.04)
Tumbes	**1.38 (1.19 to 1.60)**	**1.36 (1.18 to 1.57)**	**1.24 (1.08 to 1.43)**	**1.30 (1.12 to 1.50)**
Prediabetes (vs normal)				
Lima	1 (Reference)	1 (Reference)	1 (Reference)	1 (Reference)
Urban Puno	0.81 (0.65 to 1.02)	**0.76 (0.60 to 0.98)**	**0.77 (0.61 to 0.99)**	0.80 (0.63 to 1.01)
Rural Puno	**0.46 (0.34 to 0.61)**	**0.58 (0.42 to 0.79)**	**0.58 (0.42 to 0.80)**	**0.66 (0.49 to 0.91)**
Tumbes	**1.26 (1.07 to 1.48)**	**1.39 (1.18 to 1.64)**	**1.38 (1.16 to 1.63)**	**1.36 (1.16 to 1.61)**
Type 2 diabetes (vs normal)				
Lima	1 (Reference)	1 (Reference)	1 (Reference)	1 (Reference)
Urban Puno	1.25 (0.85 to 1.84)	1.34 (0.88 to 2.05)	1.42 (0.94 to 2.14)	1.43 (0.95 to 2.15)
Rural Puno	**0.48 (0.29 to 0.82)**	0.61 (0.35 to 1.06)	0.64 (0.37 to 1.12)	0.75 (0.42 to 1.31)
Tumbes	**1.89 (1.40 to 2.56)**	**2.04 (1.49 to 2.80)**	**1.89 (1.37 to 2.57)**	**1.93 (1.40 to 2.66)**

Estimates in bold are significant (p<0.05).

Model 1: crude model.

Model 2: adjusted by age, sex, education level and socioeconomic status.

Model 3: as model 2 plus adjustment by parental history of hypertension/diabetes.

Model 4: as model 3 plus adjustment by obesity status.

### Prevalence of main modifiable factors

Overall prevalence of daily smoking, intake of 5+ fruits/vegetables portions and heavy alcohol drinking were below 5%. Almost one-third of the study population had low levels of leisure time and transport-related physical activity, whereas 42.7% reported watching TV two or more hours per day. Finally, the overall prevalence of obesity was 26.9%. As shown in table 1, these factors were statistically different when compared by study site.

When evaluating the pattern of aggregation of risk factors, only 1.2% (38/3213) had no risk factors studied, 29.1% (935/3213) had one risk factor, 39.4% (1267/3213) had two risk factors, 23.6% (758/3213) had three risk factors and 6.7% (215/3213) had four or more risk factors.

### Association between cardiometabolic outcomes and modifiable factors

Of the six main modifiable factors studied, and after adjusting for potential confounders, both heavy alcohol drinking and obesity were positively associated with prehypertension. In addition, there was evidence that daily smoking, heavy alcohol drinking and obesity were associated with higher prevalence of hypertension. On the other hand, only obesity was positively associated with prediabetes and diabetes (table 3).

**Table 3:** Modifiable risk factors associated with hypertension and diabetes: overall crude and adjusted estimates using multinomial logistic regression

	Crude model	Adjusted model*	Crude model	Adjusted model*
	PR (95% CI)	PR (95% CI)	PR (95% CI)	PR (95% CI)
	**Prehypertension**		**Prediabetes**	
Daily smoking (yes vs no)	1.09 (0.77 to 1.53)	0.95 (0.69 to 1.31)	0.97 (0.64 to 1.49)	0.82 (0.54 to 1.24)
Heavy alcohol drinking (yes vs no)	**1.44 (1.17 to 1.78)**	**1.30 (1.06 to 1.60)**	1.10 (0.81 to 1.50)	1.14 (0.83 to 1.55)
Leisure and transport-related PA (moderate/high vs low)	0.99 (0.87 to 1.14)	0.97 (0.85 to 1.12)	0.88 (0.75 to 1.02)	1.00 (0.84 to 1.19)
Daily hours watching TV (2+ vs <2 h)	1.01 (0.90 to 1.15)	1.07 (0.94 to 1.21)	**1.26 (1.09 to 1.46)**	1.09 (0.94 to 1.27)
Fruits and vegetables intake (5+ vs <5 servings/day)	0.72 (0.49 to 1.06)	0.85 (0.58 to 1.25)	0.74 (0.47 to 1.17)	0.75 (0.48 to 1.17)
Obesity (BMI ≥30 kg/m^2^)	1.11 (0.96 to 1.27)	**1.33 (1.16 to 1.53)**	**2.03 (1.76 to 2.35)**	**1.84 (1.59 to 2.13)**
	**Hypertension**		**Diabetes**	
Daily smoking (yes vs no)	**2.13 (1.39 to 3.26)**	**1.76 (1.23 to 2.53)**	1.44 (0.54 to 3.84)	1.19 (0.46 to 3.07)
Heavy alcohol drinking (yes vs no)	**1.55 (1.01 to 2.39)**	**1.61 (1.08 to 2.40)**	0.66 (0.21 to 2.06)	0.74 (0.24 to 2.32)
Physical activity (moderate/high vs low)	0.87 (0.68 to 1.11)	1.07 (0.83 to 1.37)	**0.49 (0.32 to 0.75)**	0.75 (0.47 to 1.21)
Daily hours watching TV (2+ vs <2 h)	0.86 (0.67 to 1.09)	1.03 (0.81 to 1.31)	1.45 (0.95 to 2.20)	1.33 (0.88 to 2.01)
Fruits and vegetables intake (5+ vs <5 servings /day)	**0.31 (0.10 to 0.93)**	0.41 (0.14 to 1.23)	1.18 (0.44 to 3.15)	1.54 (0.57 to 4.15)
Obesity (BMI ≥30 kg/m^2^)	**1.43 (1.12 to 1.84)**	**2.06 (1.60 to 2.65)**	**2.58 (1.70 to 3.94)**	**2.26 (1.48 to 3.45)**

Estimates in bold are significant (p<0.05). Participants aware of hypertension/diabetes diagnosis were excluded from the analysis accordingly.

*Adjusted by sex, age, education level, socioeconomic status and study site, using robust SEs.

BMI, body mass index; PA, physical activity; PR, prevalence ratio.

**Figure 1:**
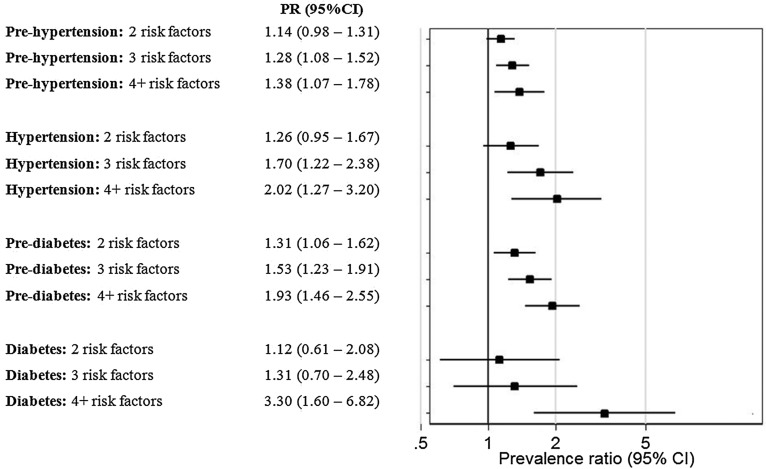
Forest plot of the association between outcomes of interest and number of modifiable risk factors. Participants aware of hypertension or diabetes diagnosis were excluded from the analysis accordingly. *Prevalence ratios (PR) were adjusted by sex, age, education level and socioeconomic status. Study site was included in the model as cluster with robust SEs.

When the cumulative effect of risk factors was evaluated, there was a clear increasing trend in the prevalence of prehypertension and hypertension with increased number of risk factors. A similar pattern was observed for prediabetes and diabetes (figure 1).

### Population attributable risks of the main modifiable factors

In the case of hypertension, PAR of daily smoking and heavy alcohol drinking were low, 3% and 2.7%, respectively; whereas PAR of low fruits and vegetable intake, and obesity, were high, 57.9% and 15.7%, respectively. In the case of diabetes, PAR of smoking was low (0.8%), whereas PAR of low levels of leisure time and transport-related physical activity (12%), daily watching TV (12.5%) and obesity (23.9%), were high.

Additionally, in the four study settings, obesity was consistently the leading risk factor for hypertension, ranging from 9.5% in rural Puno to 24.1% in urban Puno; and for diabetes, from 2.4% in rural Puno to 61.2% in Lima. See details in online supplementary (E-table S1).

## Discussion

### Main findings

Our findings signal the heterogeneity and risk factors of hypertension and type-2 diabetes prevalence within resource-constrained settings in a Latin American country. Moreover, this heterogeneity was also present when using population-attributable risk calculations. Our results, taken with the mixed geographical characteristics of Peru, which include high altitude and sea-level settings together with rural, semiurban and highly-urbanised areas, suggest the need to appropriately understand the epidemiology of cardiometabolic risk factors between and within countries. These findings contribute towards a better understanding of the diversity of disease burden, which is needed to establish appropriate interventions to tackle NCDs.

### Settings and prevalence of hypertension and type-2 diabetes

Prevalence of our main cardiometabolic outcomes was very heterogeneous and not uniformly distributed as expected. For example, given the degree of socioeconomic development and urbanisation of Lima, a priori it would be expected that all other settings should have lower rates of hypertension and diabetes than Peru's capital. Yet our results showed that, after controlling for potential confounders, individuals residing in Tumbes had 18% more prehypertension and 30% more hypertension. On the other hand, rural Puno had 28% more prehypertension rates but 19% less hypertension, though the latter was not significant. In the case of glucose metabolism status, Tumbes had greater prevalence of prediabetes (36% more) and type-2 diabetes (93% more) than Lima.

Potential explanations for these findings can be related to differential progression and staging within the epidemiological and nutrition transition, even within the same country. Peru is currently considered an upper-middle-income country, and more than 40% of total years of life lost due to premature mortality is because of NCDs.^19^ Peru's diverse geography along with unequal societal development accounts for different stages of the epidemiological and nutrition transition in different populations.^20–23^ Similar settings, especially in the Latin American region, might face similar challenges. Moreover, although a clear relationship between urbanicity and common modifiable risk factors for chronic disease has been reported,^24^ including mortality for cardiovascular events^25^; to a certain extent, the differences observed between urban and rural settings in our study may not be completely explained by urbanisation. As a result, the need of understanding the burden of NCDs profiles, including within-country and between-country heterogeneity, should be encouraged.

### Modifiable risk factors associated with hypertension/diabetes

Prevalence of daily smoking and heavy alcohol drinking was low, while almost a third of the study population had low levels of leisure time and transport-related physical activity and obesity. The consumption of fruits and vegetables according to the WHO recommendation was low. Similar trends have been reported in other developing countries. In rural settings in Nigeria, abdominal obesity, physical inactivity, smoking and alcohol consumption rates were 38.5%, 29.8%, 2.9% and 1%, respectively.^26^

Regarding hypertension, our results are similar to those previously reported in developing countries. For example, in rural Zambia, BMI was independently associated with hypertension, and so was smoking.^27^ In Vietnam, there was higher prevalence of hypertension in overweight men and alcohol drinkers.^28^ In Nepal, patients with hypertension are less physically active when compared to non-hypertensive people.^29^ Diet also matters; in China, people with a western diet have a higher likelihood of hypertension.^30^

Our results regarding diabetes and modifiable risk factors are similar to previous reports in developing countries. A study in Lebanon reported that being obese was associated with higher odds of diabetes, though vigorous physical activity had the opposite impact^31^; in addition, there was almost half the number of people with diabetes in comparison to healthy people, who were physically active.^32^ In Nepal, people with diabetes, in comparison to their healthy peers, are less physically active.^29^ Abdominal obesity is also associated with higher odds of elevated plasma glucose, as seen in females in rural Uganda.^33^

In addition, the cluster of different risk factors within a given subject is also a concern. In an urban setting in Sri Lanka, 23% had two or more risk factors, among which were physical inactivity, central obesity, family history of type-2 diabetes and BMI.^34^ In Malaysia, a national survey revealed that 14% of the population had three or more risk factors: hyperglycaemia, hypertension, hypercholesterolaemia and central obesity.^35^ Our findings, based on modifiable risk factors, showed that more than two-thirds of participants had two or more of our selected modifiable risk factors.

Despite similarities between countries, Peru has a unique profile regarding low levels of tobacco consumption: less than 5% of the participants reported daily smoking, and this value was even lower in the rural setting. Although the prevalence of smoking is low in our context, deleterious effects are well known. Most studies of NCDs have been constrained to one rural versus one urban study site, thus limiting the assessment of potential within-country heterogeneities. Moreover, reports of NCDs in high-altitude rural settings are very scarce. Under these circumstances, our study expands on better characterisation of the burden of disease in resource-constrained settings to appropriately implement strategies.

### Population attributable risks

Not all well-known modifiable risk factors were associated with our outcomes of interest. Despite the cross-sectional nature of our study, our results suggest that obesity might play an important role in developing hypertension and diabetes. Obesity, assessed by BMI, was the leading factor associated with both hypertension and diabetes, and was the same for prehypertension and prediabetes. Nevertheless, interpretation needs to be cautious, as PAR will represent the proportion of prevalent disease cases that can be attributed to exposure.^36^ Thus, although our results are compatible with the nutritional transition that low-income and middle-income countries are going through, further longitudinal studies are needed to corroborate our findings.

### Strengths and limitations

Major strengths of this study include its population-based design, the diverse geographical settings involved and the random selection of participants as well as the objective measurement of outcomes using standardised techniques. However, this study also has limitations. First, this study was not nationally representative and, thus, the inclusion of other different settings, for example, Amazonian rainforest settings, was not possible. Second, the cross-sectional nature of the study prevents establishing a causal link. However, selected variables have been reported as risk factors for developing cardiometabolic outcomes. Third, temporality and reverse causality can be an issue despite excluding those aware of hypertension or diabetes from PR and PAR calculations. Fourth, misclassification could arise, especially in the exposure variables, as many of them (ie, smoking, alcohol use, fruits and vegetables intake) were self-reported. Nevertheless, we used standardised questionnaires, including WHO STEPs, to be consistent in measure. Finally, there are inherent assumptions that need to be accounted for in PAR calculations, such as the use of data from individuals with prevalent disease instead of incident cases, and unmeasured confounding, among others.^36^ ^37^

## Conclusions

Within Peru, an evident heterogeneity in the profile of hypertension and diabetes is described. Notoriously, in addition to the diversity of patterns, the prevalence of prehypertension and prediabetes was also high across sites, signalling major future challenges for disease prevention. Our results emphasise the need for a better understanding of the epidemiology of cardiometabolic conditions to appropriately implement interventions to tackle the burden of NCDs.

**What is already known on the subject**

- Reducing the prevalence of risk factors such as smoking and alcohol consumption, among others, would contribute to reducing mortality due to cardiovascular disease and diabetes.
- Globally, high-blood pressure is a major leading risk factor for mortality, and mortality related to glucose metabolism disorders has increased rapidly.

**What this study adds**

- There is evident heterogeneity in the profile of hypertension and diabetes in different settings in Peru.
- Aggregation of national estimates of disease burden can blur the profile of cardiometabolic markers, including hypertension and diabetes, which can vary widely within a country. Understanding of these variations is needed to establish appropriate interventions.

## Supplementary Material

Web supplement

## References

[1] Global Burden of Metabolic Risk Factors for Chronic Diseases Collaboration. Cardiovascular disease, chronic kidney disease, and diabetes mortality burden of cardiometabolic risk factors from 1980 to 2010: a comparative risk assessment. Lancet Diabetes Endocrinol 2014;2:634–47. 10.1016/S2213-8587(14)70102-0 24842598 PMC4572741

[2] Murray CJ, Vos T, Lozano R, et al Disability-adjusted life years (DALYs) for 291 diseases and injuries in 21 regions, 1990–2010: a systematic analysis for the Global Burden of Disease Study 2010. Lancet 2012;380:2197–223. 10.1016/S0140-6736(12)61689-4 23245608

[3] Kontis V, Mathers CD, Rehm J, et al Contribution of six risk factors to achieving the 25×25 non-communicable disease mortality reduction target: a modelling study. Lancet 2014;384:427–37. 10.1016/S0140-6736(14)60616-4 24797573

[4] Bloom DE, Cafiero ET, Jane-Llopis E, et al The global economic burden of noncommunicable diseases. Geneva, Switzerland: World Economic Forum, 2011.

[5] Lall D, Prabhakaran D Organization of primary health care for diabetes and hypertension in high, low and middle income countries. Expert Rev Cardiovasc Ther 2014;12:987–95. 10.1586/14779072.2014.928591 24934722

[6] Ebrahim S, Pearce N, Smeeth L, et al Tackling non-communicable diseases in low- and middle-income countries: is the evidence from high-income countries all we need?. PLoS Med 2013;10:e1001377 10.1371/journal.pmed.1001377 23382655 PMC3558465

[7] Miranda JJ, Herrera VM, Chirinos JA, et al Major cardiovascular risk factors in Latin America: a comparison with the United States. The Latin American Consortium of Studies in Obesity (LASO). PloS ONE 2013;8:e54056 10.1371/journal.pone.0054056 23349785 PMC3547948

[8] Miranda JJ, Bernabe-Ortiz A, Smeeth L, et al Addressing geographical variation in the progression of non-communicable diseases in Peru: the CRONICAS cohort study protocol. BMJ Open 2012;2:e000610. 10.1136/bmjopen-2011-000610 PMC3278488 22240652

[9] Chobanian AV, Bakris GL, Black HR, et al Seventh report of the Joint National Committee on Prevention, Detection, Evaluation, and Treatment of High Blood Pressure. Hypertension 2003;42:1206–52. 10.1161/01.HYP.0000107251.49515.c2 14656957

[10] American Diabetes Association. Diagnosis and classification of diabetes mellitus. Diabetes Care 2014;37(Suppl 1):S81–90. 10.2337/dc14-S081 24357215

[11] Hallal PC, Gomez LF, Parra DC, et al Lessons learned after 10 years of IPAQ use in Brazil and Colombia. J Phys Act Health 2010;7(Suppl 2):S259–64. 20702914 10.1123/jpah.7.s2.s259

[12] World Health Organization and Food and Agriculture of the United Nations. Fruit and vegetables for health. Report of a Joint FAO/WHO workshop Geneva, Switzerland, 2005.

[13] World Health Organization. Reducing risks, promoting healthy life. Geneva, Switzerland, 2002.

[14] Alberti KG, Eckel RH, Grundy SM, et al Harmonizing the metabolic syndrome: a joint interim statement of the International Diabetes Federation Task Force on Epidemiology and Prevention; National Heart, Lung, and Blood Institute; American Heart Association; World Heart Federation; International Atherosclerosis Society; and International Association for the Study of Obesity. Circulation 2009;120:1640–5. 10.1161/CIRCULATIONAHA.109.192644 19805654

[15] Ahmad OB, Boschi-Pinto C, Lopez AD, et al Age standardization of rates: a new WHO standard. Geneva, Switzerland: WHO, 2001.

[16] Coutinho LM, Scazufca M, Menezes PR Methods for estimating prevalence ratios in cross-sectional studies. Rev Saude Publica 2008;42:992–8. 10.1590/S0034-89102008000600003 19009156

[17] Newson RB Attributable and unattributable risks and fractions and other scenario comparisons. Stata J 2013;13:672–98.

[18] Greenland S, Drescher K Maximum likelihood estimation of the attributable fraction from logistic models. Biometrics 1993;49:865–72. 10.2307/2532206 8241375

[19] Perel P, Miranda JJ, Ortiz Z, et al Relation between the global burden of disease and randomized clinical trials conducted in Latin America published in the five leading medical journals. PloS ONE 2008;3:e1696 10.1371/journal.pone.0001696 18301772 PMC2246037

[20] Goldstein J, Jacoby E, del Aguila R, et al Poverty is a predictor of non-communicable disease among adults in Peruvian cities. Prev Med 2005;41:800–6. 10.1016/j.ypmed.2005.06.001 16061280

[21] Popkin BM, Adair LS, Ng SW Global nutrition transition and the pandemic of obesity in developing countries. Nutr Rev 2012;70:3–21. 10.1111/j.1753-4887.2011.00456.x 22221213 PMC3257829

[22] Yusuf S, Ounpuu S Tackling the growing global burden of atherosclerotic cardiovascular diseases. Eur J Cardiovasc Prev Rehabil 2003;10:236–9. 10.1097/00149831-200308000-00003 14555877

[23] Pan American Health Organization. Health in Americas—2012 edition. Washington DC, US: PAHO, 2012.

[24] Allender S, Wickramasinghe K, Goldacre M, et al Quantifying urbanization as a risk factor for noncommunicable disease. J Urban Health 2011;88:906–18. 10.1007/s11524-011-9586-1 21638117 PMC3191205

[25] Burroughs Pena MS, Bernabe-Ortiz A, Carrillo-Larco RM, et al Migration, urbanisation and mortality: 5-year longitudinal analysis of the PERU MIGRANT study. J Epidemiol Community Health 2015;69:715–18. 10.1136/jech-2015-205657 25987723 PMC4494660

[26] Ogunmola OJ, Olaifa AO, Oladapo OO, et al Prevalence of cardiovascular risk factors among adults without obvious cardiovascular disease in a rural community in Ekiti State, Southwest Nigeria. BMC Cardiovasc Disord 2013;13:89 10.1186/1471-2261-13-89 24138186 PMC4016363

[27] Mulenga D, Siziya S, Rudatsikira E, et al District specific correlates for hypertension in Kaoma and Kasama rural districts of Zambia. Rural Remote Health 2013;13:2345. 24050622

[28] Do HT, Geleijnse JM, Le MB, et al National prevalence and associated risk factors of hypertension and prehypertension among Vietnamese adults. Am J Hypertens 2015;28:89–97. 10.1093/ajh/hpu092 24862960

[29] Vaidya A, Krettek A Physical activity level and its sociodemographic correlates in a peri-urban Nepalese population: a cross-sectional study from the Jhaukhel-Duwakot health demographic surveillance site. Int J Behav Nutr Phys Act 2014;11:39 10.1186/1479-5868-11-39 24628997 PMC3984675

[30] Sun J, Buys NJ, Hills AP Dietary pattern and its association with the prevalence of obesity, hypertension and other cardiovascular risk factors among Chinese older adults. Int J Environ Res Public Health 2014;11:3956–71. 10.3390/ijerph110403956 24727356 PMC4025020

[31] Costanian C, Bennett K, Hwalla N, et al Prevalence, correlates and management of type 2 diabetes mellitus in Lebanon: findings from a national population-based study. Diabetes Res Clin Pract 2014;105:408–15. 10.1016/j.diabres.2014.06.005 25005850

[32] Sibai AM, Costanian C, Tohme R, et al Physical activity in adults with and without diabetes: from the ‘high-risk’ approach to the ‘population-based’ approach of prevention. BMC Public Health 2013;13:1002 10.1186/1471-2458-13-1002 24153099 PMC4015809

[33] Maher D, Waswa L, Baisley K, et al Distribution of hyperglycaemia and related cardiovascular disease risk factors in low-income countries: a cross-sectional population-based survey in rural Uganda. Int J Epidemiol 2011;40:160–71. 10.1093/ije/dyq156 20926371 PMC3043279

[34] Wijesuriya M, Gulliford M, Charlton J, et al High prevalence of cardio-metabolic risk factors in a young urban Sri-Lankan population. PloS ONE 2012;7:e31309 10.1371/journal.pone.0031309 22348068 PMC3278419

[35] Selvarajah S, Haniff J, Kaur G, et al Clustering of cardiovascular risk factors in a middle-income country: a call for urgency. Eur J Prev Cardiol 2013;20:368–75. 10.1177/2047487312437327 22345688

[36] Benichou J A review of adjusted estimators of attributable risk. Stat Methods Med Res 2001;10:195–216. 10.1191/096228001680195157 11446148

[37] Gefeller O Comparison of adjusted attributable risk estimators. Stat Med 1992;11:2083–91. 10.1002/sim.4780111606 1293670

