# Supplementary material for: Contribution of modifiable risk factors for hypertension and type-2 diabetes in Peruvian resource-limited settings
Source: J Epidemiol Community Health. 2015 Aug 6;70(1):49–55. doi: 10.1136/jech-2015-205988 (PMC4717378; doi:10.1136/jech-2015-205988)

ONLINE SUPPLEMENT

E-Table 1: Population attributable risks of the main modifiable risk factors

	Total			Lima			Urban Puno			Rural Puno			Tumbes		
	**P**	**PR**	**PAR**	**P**	**PR**	**PAR**	**P**	**PR**	**PAR**	**P**	**PR**	**PAR**	**P**	**PR**	**PAR**
**Hypertension**															
Daily smoking	3.2%	**1.76**	3.0%	3.2%	**2.61**	6.0%	2.1%	--	--	0.2%	--	--	5.4%	1.39	3.1%
Heavy alcohol drinking	5.3%	**1.61**	2.7%	5.5%	1.36	1.5%	6.5%	1.03	0.1%	2.9%	1.82	2.3%	5.8%	1.72	4.6%
Low physical activity	31.9%	0.94	-2.3%	19.1%	1.20	3.7%	21.3%	0.53	-10.6%	25.6%	0.99	-2.7%	54.2%	0.86	-8.0%
2+ hours watching TV	42.7%	1.03	1.1%	48.4%	1.07	3.3%	46.2%	1.13	4.7%	14.3%	0.86	-1.4%	50.9%	1.02	1.1%
Low fruits and vegetables intake	95.9%	2.42	57.9%	93.3%	1.05	4.2%	93.4%	--	--	98.1%	--	--	98.7%	--	--
Obesity	26.9%	**2.06**	15.7%	31.7%	**2.22**	22.1%	26.6%	**2.52**	24.1%	10.1%	**2.74**	9.5%	31.6%	**1.68**	12.5%
**Diabetes**															
Daily smoking	3.2%	1.19	0.8%	3.2%	1.42	1.7%	2.1%	--	--	0.2%	--	--	5.4%	1.39	1.8%
Heavy alcohol drinking	5.3%	0.74	-1.3%	5.5%	2.33	6.7%	6.5%	--	--	2.9%	--	--	5.8%	0.47	-2.4%
Low physical activity	31.9%	1.33	12.0%	19.1%	1.01	0.2%	21.3%	2.19	19.8%	25.6%	1.29	7.5%	54.2%	1.35	17.0%
Daily watching TV (2+ hours)	42.7%	1.33	12.5%	48.4%	1.51	19.8%	46.2%	1.19	7.2%	14.3%	--	--	50.9%	1.45	18.2%
Low fruits and vegetables intake	95.9%	0.65	-51.5%	93.3%	0.55	-71.7%	93.4%	0.68	-42.6%	98.1%	--	--	98.7%	0.51	-95.0%
Obesity	26.9%	**2.26**	23.9%	31.7%	**7.50**	61.2%	26.6%	1.59	10.1%	10.1%	1.32	2.4%	31.6%	**1.78**	19.0%

P = prevalence, PR = prevalence ratio, PAR = population attributable risk\
Bolded estimates are significant (p<0.05)

E-Figure 1: Study participants flowchart, all sites

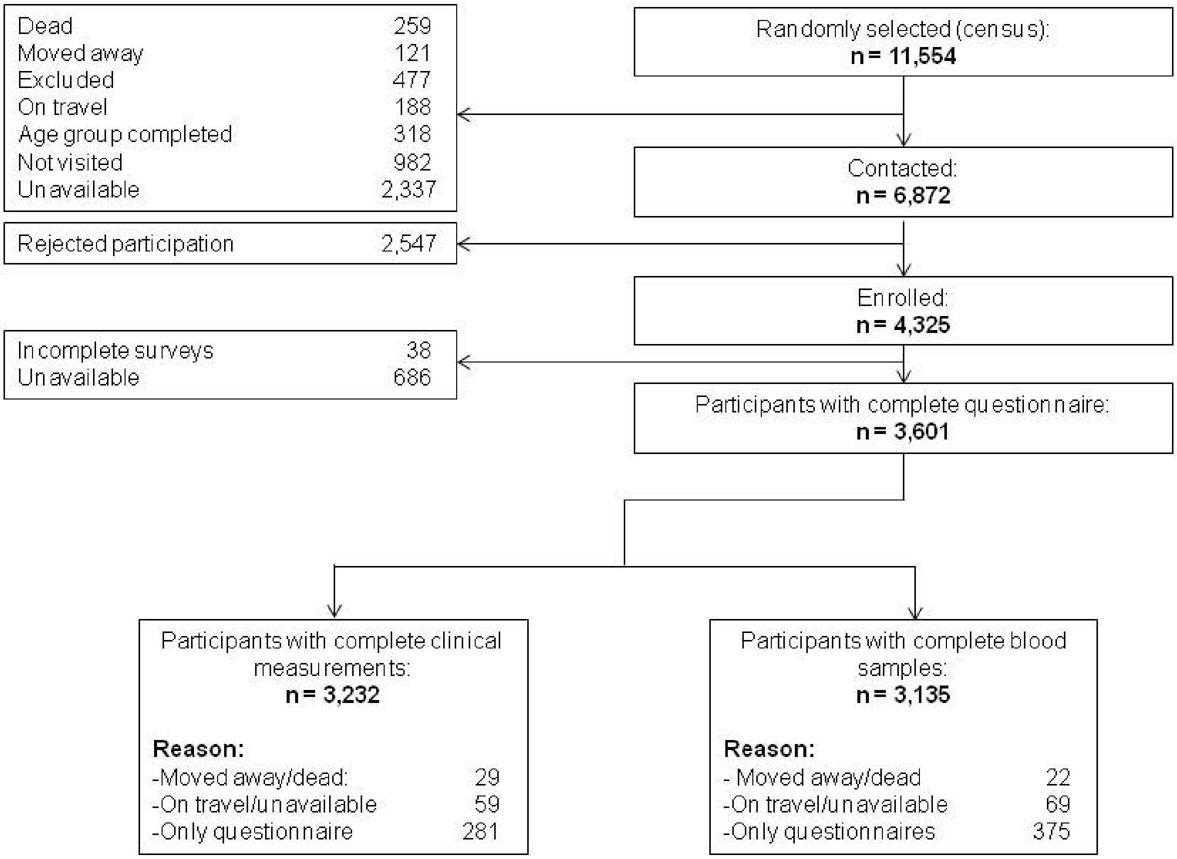

Supplement: Web supplement [file jech-2015-205988-s1.pdf]
